# Deep Insights into the Specific Evolution of Fungal Hybrid B Heme Peroxidases

**DOI:** 10.3390/biology11030459

**Published:** 2022-03-17

**Authors:** Marcel Zámocký, Miloš Musil, Maksym Danchenko, Peter Ferianc, Katarína Chovanová, Peter Baráth, Andrej Poljovka, David Bednář

**Affiliations:** 1 Laboratory for Phylogenomic Ecology, Institute of Molecular Biology, Slovak Academy of Sciences, Dúbravská cesta 21, SK-84551 Bratislava, Slovakia; peter.ferianc@savba.sk (P.F.); katarina.chovanova@savba.sk (K.C.); andrej.poljovka@savba.sk (A.P.); 2 University of Natural Resources and Life Sciences, Vienna, Department of Chemistry, Institute of Biochemistry, Muthgasse 18, 1190 Vienna, Austria; 3 Loschmidt Laboratories, Department of Experimental Biology and RECETOX, Faculty of Science, Masaryk University, CZ-61137 Brno, Czech Republic; imusilm@fit.vutbr.cz (M.M.); 222755@mail.muni.cz (D.B.); 4 International Clinical Research Centre, St. Anne’s University Hospital Brno, CZ-65691 Brno, Czech Republic; 5 Department of Information Systems, Faculty of Information Technology, Brno University of Technology, CZ-61200 Brno, Czech Republic; 6 Department of Glycobiology, Institute of Chemistry, Slovak Academy of Sciences, Dúbravská cesta 9, SK-84538 Bratislava, Slovakia; maksym.danchenko@savba.sk (M.D.); peter.barath@savba.sk (P.B.)

**Keywords:** hybrid B heme peroxidase, peroxidase–catalase superfamily, oxidative stress, enzymatic antioxidant, ancestral sequence reconstruction

## Abstract

**Simple Summary:**

Fungi are well equipped to cope with oxidative stress and the reactive oxygen species that are, in the case of phytopathogens, produced mainly by the plant host for defence purposes. Peroxidases represent the major line of evolution for rapid decomposition of harmful peroxides in all aerobically metabolising organisms. In all the sequenced fungal genomes, many divergent genes coding for various peroxidases have been discovered, and Hybrid B heme peroxidases represent a distinctive mode of fungal-gene evolution within a large peroxidase–catalase superfamily that ranges from bacteria to plants.

**Abstract:**

In this study, we focus on a detailed bioinformatics analysis of *hyBpox* genes, mainly within the genomes of *Sclerotiniaceae* (Ascomycota, Leotiomycetes), which is a specifically evolved fungal family of necrotrophic host generalists and saprophytic or biotrophic host specialists. Members of the genus *Sclerotium* produce only sclerotia and no fruiting bodies or spores. Thus, their physiological role for peroxidases remains open. A representative species, *S. cepivorum,* is a dangerous plant pathogen causing white rot in *Allium* species, particularly in onions, leeks, and garlic. On a worldwide basis, the white rot caused by this soil-borne fungus is apparently the most serious threat to *Allium*-crop production. We have also found very similar peroxidase sequences in the related fungus *S. sclerotiorum*, although with minor yet important modifications in the architecture of its active centre. The presence of *ScephyBpox1-*specific mRNA was confirmed by transcriptomic analysis. The presence of Hybrid B peroxidase at the protein level as the sole extracellular peroxidase of this fungus was confirmed in the secretome of *S. cepivorum* through detailed proteomic analyses. This prompted us to systematically search for all available genes coding for Hybrid B heme peroxidases in the whole fungal family of *Sclerotiniaceae*. We present here a reconstruction of their molecular phylogeny and analyse the unique aspects of their conserved-sequence features and structural folds in corresponding ancestral sequences.

## 1. Introduction

During their unique evolution in various environments, fungi were adapted mainly for aerobic metabolism and are mostly well equipped with efficient antioxidants [1], the most important of them being enzymatic [2]. These rather divergent proteins help them to cope with the harmful or even deleterious effects that can be caused by numerous reactive oxygen species. ROS emerge either as by-products of their aerobic catabolism or are present in the fungal environment. Three main groups of oxidoreductases were described as efficient antioxidants: superoxide dismutases, catalases, and peroxidases. Through the tight regulation of their genes and corresponding RNA transcripts, they have an impact on cellular redox homeostasis in numerous organisms [3,4].

The filamentous ascomycetous fungus *Sclerotium cepivorum* Berk is a typical phytopathogenic representative and, more specifically, it is a plant necrotroph [5]. It causes a devastating white rot in several *Allium* species, particularly onions, leeks, and garlic by producing a characteristic strong colour changes [6]. This can be considered a serious plant disease and a large threat to worldwide allium production that needs to be prevented [7]. Efficient measures need to be taken based mainly on the detailed knowledge of its genetic diversity [8].

The complete genome of *Sclerotium cepivorum* was released previously, [9] showing the presence of up to 11,130 protein-coding genes. However, still less is known about the antioxidant machinery of this dangerous plant-pathogenic fungus against various forms of oxidative stress. Oxidative burst, in general, can be exploited by the attacked plant for efficient defence against invading necrotrophic fungi; therefore, the detailed investigation of enzymatic antioxidants can also have an impact on the detailed understanding of host–pathogen interactions. We focus this study on a detailed, in silico analysis of distinctive fungal hybrid heme peroxidases. We reconstructed their molecular phylogeny and analysed their corresponding ancestors from the point of view of their structure–function relationships and their conserved sequences and structural motifs during evolution. In addition to the detailed description of particular phytopathogenic Ascomycetes representatives, we followed the outlined evolutionary history several steps back and demonstrated interesting structural features of reconstructed heme peroxidase ancestors for both Ascomycota and Basidiomycota.

## 2. Materials and Methods

### 2.1. Fungal Strain and Growth Conditions

The fungal strain *Sclerotium cepivorum*, Berkeley CBS 276.93 was obtained from Fungal Biodiversity Centre (CBS, Utrecht, The Netherlands). Only this strain was used throughout all experiments. Fungal culture was routinely grown at 25 °C in potato-dextrose-broth medium (Neogen, Lansing, MI, USA) containing 4 g potato infusion and 20 g dextrose, with the addition of fresh, sterilised onion extract, pH 5.1 for 1 L final volume. This fungus was grown either on solid agar medium (with the addition of 15 g/L agar) or in the liquid medium that was used for cultivation in 500 mL Schott flasks. In this case, the two cultures were grown in a final volume of 100 mL, without shaking, at 25 °C for 7 days, as optimised previously [10]. Afterwards, H_2_O_2_ was added to the first culture at a final concentration of 1 mM, and the second culture was grown in parallel without the addition of peroxide. Growth was continued for 30 min. at the same temperature with agitation of 130 rpm on a rotary shaker. Obtained samples were used for the isolation of DNA or RNA (Section 2.2) or for proteomic analysis (Section 2.3).

### 2.2. Isolation of Fungal DNA, Total RNA and Synthesis of cDNA

Fungal genomic DNA from 100 mg of frozen *S. cepivorum* mycelium was isolated with GeneJET Genomic DNA Purification Kit (ThermoFisher Scientific, Waltham, MA, USA). Total RNA of this fungus from the frozen mycelium of samples with and without the addition of 1 mM H_2_O_2_ was purified with RNeasy Plus Mini Kit (Qiagen, Hilden, Germany). From the obtained RNA libraries, corresponding cDNA was prepared with First Strand cDNA synthesis kit (ThermoFisher Scientific, Waltham, MA, USA) by using the random hexamers approach. Resulting cDNAs were amplified and then sequenced with the *hyBpox*-specific primers described in Table S1 at Genseq (Benešov, Czech Republic). The obtained intronless sequences were translated into corresponding HyBpox protein sequences of interest with BioEdit software version 7.2.5 [11].

### 2.3. Investigation of Fungal Secretome and Proteomic Analysis

The fungal protein secretome of *S. cepivorum* CBS 276.93 was investigated from the liquid PDB cultures obtained under conditions described in Section 2.1. After the achieved time period, the growth medium was separated from the mycelium by filtration through sterile 2R filter paper with a diameter of 150 mm (paper mill Pernštejn, Pernštejn nad Ohří, Czech Republic) under vacuum and the clear filtrate was used as a secretome. For comparison, soluble proteins extracted from the corresponding fungal mycelium were also produced using BeadBug equipment (Benchmark Scientific, Sayreville, NJ, USA). Sterile glass beads of 1.0 mm diameter were applied at maximal speed (4000 rpm) for 3 min for homogenisation of the separated fungal cells. Protein concentration was determined using a standard Bradford assay. All obtained fungal proteins (extracellular and intracellular) were precipitated using trichloroacetic acid (TCA, Merck, Kenilworth, NJ, USA) with the method described in [12]. Obtained pellets of purified cellular and secreted proteins were solubilised with 8 M urea in 100 mM TEAB buffer of pH 8.5 (both Merck, Kenilworth, NJ, USA). Upon quantification by standard Bradford assay, 50 µg aliquots were reduced with dithiothreitol and alkylated with iodoacetamide (both Merck, Kenilworth, NJ, USA). Consequently, proteins were digested overnight with trypsin, and peptides were purified with C18 spin columns. Peptide concentration was verified by fluorometric assay. Liquid-chromatography-coupled mass spectrometry (LC–HRMS) analysis was performed as described earlier [13]. Briefly, 750 ng of purified peptides were loaded onto a trap column (PepMap100 C18, 300 μm × 5 mm, 5 μm particle size, Dionex, Waltham, MA, USA) and separated with an EASY-Spray C18 analytical column (75 μm × 500 mm, 5 μm particle size, Thermo Fisher Scientific, Waltham, MA, USA) in Ultimate 3000 RSLCnano system (Dionex) in a 120 min gradient of 2–34% acetonitrile supplemented with 0.1% formic acid at a flow rate of 250 nL/min. Eluted peptides were sprayed into an Orbitrap Elite mass spectrometer (Thermo Fisher Scientific, Waltham, MA, USA), and spectra were collected in the data-dependent mode using the Top15 strategy for the selection of precursor ions. Datasets were processed by MaxQuant v1.6.17.0 using standard parameters [14]. Protein and peptide identities were accepted at a 1% false discovery rate. The label-free quantification relied on sums of precursor-ion intensities. The search was performed against transcriptome-derived protein sequences (11,132 accessions). The statistical analysis was performed using Perseus v1.6.15.0. computational platform [15] After log_2_ transformation of the relative protein abundances, Student’s *t*-tests were calculated and the results were visualised as a volcano plot.

### 2.4. Multiple Sequence Alignment and Reconstruction of Evolutionary Relationships

Complete protein sequences of 170 peroxidases belonging to the peroxidase–catalase superfamily (listed in Table S2) were aligned using the MUSCLE program [16] implemented in MEGA-X suite [17]. The optimised parameters were as follows: gap open −0.8, gap extend −0.05, and hydrophobicity multiplier 0.9, and the output was inspected and manually refined with GeneDoc software (www.psc.edu/biomed/genedoc accessed on 2 February 2022). Putative signal sequences of analysed peroxidases with the detection of Sec/SPI cleavage sites were predicted with SignalP 6.0 server [18]. Phylogenetic relationships within obtained multiple sequence alignments were reconstructed using MEGA-X by choosing the maximum likelihood method. The Whelan and Goldman substitution model [19] with frequencies and invariant sites (WAG+G+I+F) was applied as the statistically proven best-substitution model revealing the lowest Bayesian information criterion. Therefore, gamma-distributed substitution rates with the presence of invariant sites and 4 categories (+G, parameter = 1.6099) were chosen for optimal phylogeny reconstruction. Partial deletion with site coverage cut off at 90% and a very strong branch-swap filter were selected. This means that 309 alignment positions were used for this phylogenetic reconstruction and 1000 bootstrap replicates were applied to them.

### 2.5. Ancestral Sequence Reconstruction for Peroxidases

Ancestral sequences in important nodes of the obtained evolutionary tree for a predefined set of 170 heme peroxidases (Section 2.4 and Table S2) were reconstructed using parts of the typical workflow adapted from the FireProt^ASR^ web server [20]. FireProt^ASR^ provides users with a fully automatised calculation of ancestral sequences, including searches for sequence homologs for particular query sequences. However, as the homolog search and construction of the multiple sequence alignment and the phylogenetic tree were already completed within the MEGA-X suite, only the third stage of the standard FireProt^ASR^ calculation was utilised. The best fit for a given set of homolog sequences, namely also WAG, was applied as retrieved by IQ-TREE [21] and the phylogenetic reconstruction was verified independently with RAxML [22]. Ancestral sequences of Hybrid B heme peroxidases were calculated using the PAML module [23] implemented in the LAZARUS software [24]. Finally, based on the tree topology, branch lengths, and the posterior probabilities obtained from LAZARUS, the algorithm in FireProt^ASR^ was employed to reconstruct all ancestral gaps [20].

### 2.6. Modelling of 3D Structures for Ancestral Heme Peroxidases

Three-dimensional models for the structures of selected present-day as well as ancestral heme peroxidases in six predecessor nodes were obtained using the AlphaFold 2 [25]. The prosthetic heme group was placed in the binding pocket by aligning the active centres of the modelled structures with the closest available crystal structure (PDB ID: 5ABQ) and transforming the heme group from the crystal to the model. Modelled present-day and ancestral peroxidases with docked heme groups were then optimised using the 3DRefine server [26].

### 2.7. Identification of Protein Tunnels and Ligand Docking

Tunnels in the protein were identified using the Caver 3.0.3 [27] plugin integrated into the PyMol 2.3.0. (https://www.pymol.org/pymol accessed on 2 February 2022). The minimum probe radius was set to 0.7 to detect even the narrower tunnels present in the ScepHyBpox1 model. The remaining parameters were kept at their default settings. The coordinates of the starting point of the tunnel search were calculated using the Arg69 and His73 residues together with the heme cofactor. The AutoDock Vina plugin [28] was then utilised to dock two typical ligands, hydrogen peroxide (PubChem CID: 784) and guaiacol (PubChem CID: 460), into the active site of the protein. The active site was enveloped by a gridbox of size 40A using Arg69, His73, and heme cofactor to calculate its central point. Ten poses were calculated for both of the analysed ligands. This process was repeated for ScepHyBpox1 and all six of the selected ancestral sequences.

## 3. Results

### 3.1. Detection of Specific Fungal mRNA for ScepHyBpox1 and Gene Architecture

**Figure 1:**
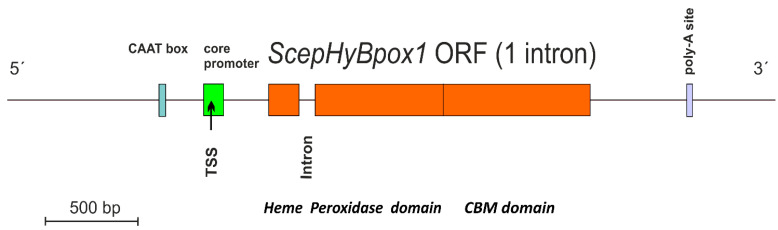
Gene architecture of *ScepHyBpox1* with detection of core promoter region (green), CAAT box (blue), and poly-A site (violet). TSS—transcription start site. The border between regions coding for two separate domains is labelled and drawn to scale according to predictions from FGENESH 2.6.

*ScephyBpox1-mRNA* was found in both fungal mRNA libraries, either induced with the addition of 1 mM hydrogen peroxide (final concentration) or also in the control sample without this addition. After performing cDNA synthesis within both independent mRNA libraries, the same *ScephyBpox1* product was obtained from the fungal strain CBS 276.93, with a total length of 1614 base pairs for the open reading frame. The presence of 1 spliced intron with a length of 54 nucleotides was detected by direct comparison of genomic and RT-PCR fragments (Figure S1) and was obvious also after pairwise sequence alignment with the corresponding region in the genomic DNA (from strain JRUF-117), available in GenBank under accession number RCTD01000019. The spliced, intronless mRNA corresponded to a protein with a total length of 537 amino acids, which is comparable with other Hybrid B heme peroxidases from *Sclerotiniaceae* (cf. Table S2). This newly discovered cDNA sequence can be found in GenBank under accession number OM395782 and the translated protein under GenBank id UMM61353. The transcription start site detected in the genomic region with FGENESH 2.6 (softberry.com accessed on 1 March 2022) was located 310 nucleotides upstream from the start codon for *ScepHyBpox1*, and within the core promoter region. The typical CAAT box upstream from the TSS was also found with this approach. Moreover, the detection of a poly-adenylation signal at the 3′ terminus of this sequence confirmed that the obtained mRNA was complete and no additional domain was encoded further downstream. In contrast, within some distantly related Hybrid B heme peroxidases the C-terminal non-heme domain can be multiplicated (e.g., in CcochHyBpox2 or CgHyBpox2—see Table S2). A complete architecture of the newly discovered SceHyBpox1 gene is presented in Figure 1.

### 3.2. Detection of Specific Protein Patterns of ScepHyBpox1 in the Fungal Secretome

**Figure 2:**
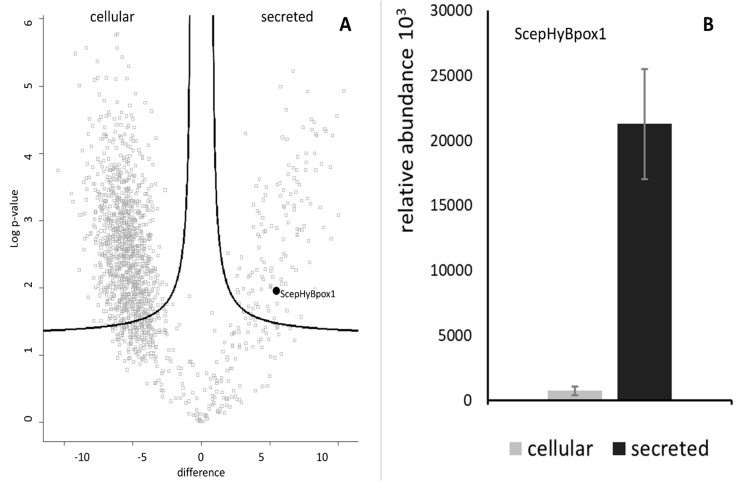
Identification and quantification of the peroxidase ScepHyBpox1 in the proteomic dataset. (**A**) The volcano plot shows the distribution of identified proteins according to their statistical significance (Log *p*-value) and magnitude of change (difference). Peroxidase ScepHyBpox1 significantly accumulated in secretome samples compared with intact cells. (**B**) Observed label-free quantitation intensities of ScepHyBpox1 protein in cellular and secreted protein samples are depicted as means with SE error bars (*n* = 3).

In the next step, a typical secretome of the filamentous fungus *S. Cepivorum* was analysed after incubation in PDB medium for 7 days at 25 °C. The results of mass spectrometric analysis are presented in Figure 2 as a comparison between the intracellular and secreted proteins. The relative abundance values for ScepHyBpox1 demonstrate that this protein was significantly accumulated in the samples collected from the secretome, as was expected for a member of this peroxidase subfamily. Even Table S3 shows that this is the sole extracellular peroxidase among the 126 quantified proteins significantly more abundant in the secretome fraction. Among many others, three signals of secreted GMC oxidoreductase are also interesting in this respect as GMC oxidoreductases, as flavin enzymes, can produce hydrogen peroxide, which is the main substrate for ScepHyBpox1. In Table S4, identification details (including the list of matched peptides visualised on the protein sequence), quantification, and statistical analysis of ScepHyBpox1 in secreted and cellular fractions are presented. Peptides reliably identified by mass spectrometry are in bold, and the sequence likely cleaved by Sec/SPI is underlined.

It can be concluded that the discovered peptide fragments are distributed throughout the whole protein sequence of this peroxidase. Moreover, in Figure S2, annotated fragmentation spectra belonging to peptides from this study identifying extracellular peroxidase ScepHyBpox1 are shown. From the presented cDNA-sequencing output and mass spectrometry analysis, it can be deduced that the protein sequence of ScepHyBpox1 identified in the strain CBS 276.93 is completely identical to the protein sequence with accession number KAF7861899 (Table S2) from the genome of a different strain, JRUF-117, where it is annotated as just a “hypothetical protein” in the automatically translated proteome from BioProject PRJNA494516 [9].

### 3.3. Evolutionary Relationships of Hybrid B Heme Peroxidases

**Figure 3:**
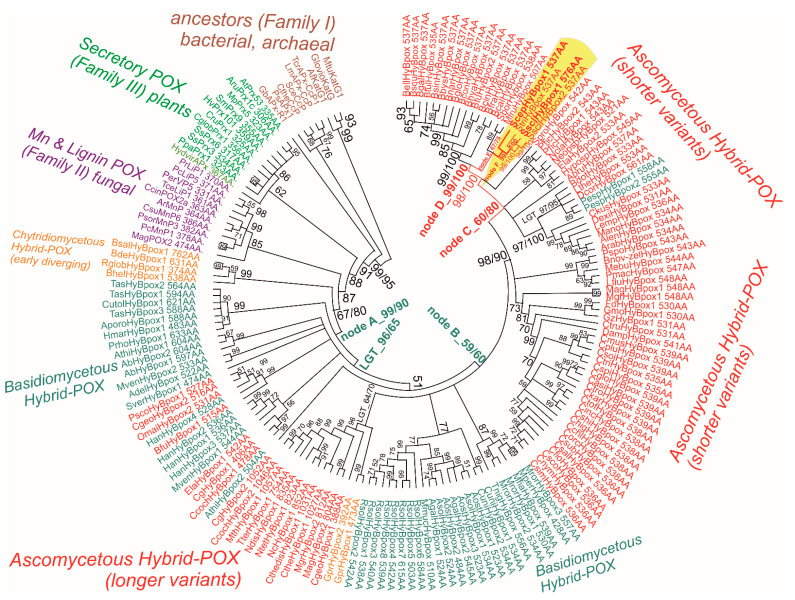
Reconstructed evolutionary tree of Hybrid B heme peroxidases obtained with the maximum likelihood method within the MEGA X suite [17]. The Whelan and Goldman amino-acid substitution model with frequencies (WAG+G+I+F) was applied for this analysis because the statistically best-proven model with the lowest BIC score and gamma-distributed substitution rates for the presence of invariant sites was applied. The bootstrap consensus tree, with site coverage cut off set at 90%, is also presented. Numbers in nodes represent bootstrap values for 1000 performed replicates obtained from MEGA ML/RAxML. (only values above 50 are shown). Abbreviations of sequences used are listed in Table S2 and correspond with RedoxiBase (for those sequences not yet available there, the same format was used).

Evolutionary relationships were reconstructed for a recently discovered peroxidase subfamily, namely for Hybrid B heme peroxidases that reveal significant differences from previously described Hybrid-A counterparts [29] from the same peroxidase–catalase superfamily. Hybrid B peroxidases are apparently longer and more complex (Table S2) with respect to their domain organisation and architecture, and in comparison with the rather prevalent short-length, single-domain heme peroxidases that occur in the majority of identified sequences within the whole peroxidase–catalase superfamily. The resulting robust phylogenetic tree for the 170 full-length protein sequences of Hybrid B peroxidases was obtained with the maximum likelihood method by applying the Whelan and Goldman substitution matrix and gamma-distributed substitution rates. For statistical purposes, 1000 bootstrap replicates were performed, and the resulting consensus tree is presented in circular form in Figure 3. This is an update to and extension of the previous work on the phylogeny of all Hybrid peroxidases [30], which is now more focused on the representatives obtained from various phytopathogenic Ascomycota.

From the upper part of this bootstrap consensus tree, on its left side, it can be followed that this particular subfamily diverged from a common ancestor that all HyBpox share with Family II (known mainly as fungal lignin and manganese peroxidases) and also shared with Family III earlier (described as plant-secretory peroxidases, an example of which is the well-known horseradish peroxidase). A diversification had occurred already within the early steps in the evolution of the distinct HyBpox subfamily; the basal clade of still-putative Hybrid B heme peroxidases is positioned among Chytridiomycetous fungi with high bootstrap support. Based on investigated microfossils, it was reported [31] that zoosporic Chytridiomycota are a real ancient fungal division, and our phylogenetic reconstruction demonstrates that Hybrid B heme peroxidases were present in the fungal kingdom from the beginning of its primordial evolution, estimated at around 750 million years ago [32]. The early evolutionary step from the basal (i.e., chytridiyomycetous) HyBpox clade led towards a specific clade of corresponding genes in tremellomycetes that already belonged to the anamorphic basidiomycetous yeasts originating in temperate and tropical environments [33]. Then, the diversification of *hyBpox* genes took its place mainly within the broadly distributed class of agaricomycetes, for which numerous highly similar gene sequences are available from recently sequenced mushroom genomes [34,35]. In some physiologically different species, such as the phytopathogenic *Heterobasidion annosum* or *Rhizoctonia solani*, multiple *hyBpox* isoforms exist (cf. Table S2), which probably arose through multiple gene-duplication events within the same fungal genome.

By further following the unique history of this subfamily in the robust evolutionary tree presented, the ascomycetous descendants that could have emerged through a lateral gene transfer event from some Basidiomycota predecessor serving as gene donor can be observed in the next clades. This is supported by a high bootstrap value (Figure 3: LGT_96/65). It is interesting to note that all the sequences of this particular clade (in the lower part of Figure 3, on the left side) presented here come from the typical ascomycetous class of mostly filamentous Soradiomycetes (Table S2). However, much longer coding sequences occured (in some cases proteins exceed the length of 1000 amino acids) in this clade, so a classical, lateral gene transfer (LGT) and also the acquisition of a novel part for this gene that was fused onto the C-teminus, namely the CBM domain(s), must have also occured. Further diversification of this unique subfamily can also be observed within the clades of Basidiomycota genus Rhizoctonia (mostly phytopathogenic fungi belonging to the class of Agaricomycetes). By following the last clades in the evolutionary tree, it is apparent that the latest descendants appear again among the ascomycetous fungi—as seen on the right side of Figure 3 in its upper part. This is the most abundant clade and shorter representatives of around 530–550 amino acids in length come mainly from the genomes of various phytopathogenic fungi from the class Leotiomycetes. Around the ScepHyBpox1 sequence (labelled Figure 3, red on a yellow background) coding for peroxidase from the abovementioned phytopathogen *Allium* species, high bootstrap support is achieved. This particular sequence of Hybrid B peroxidase from *Sclerotium cepivorum* can thus be considered a typical model protein for all closely related heme peroxidases from the mostly phytopathogenic ascomycetous class of Leotiomycetes; however, the corresponding native peroxidases, other than the newly discovered ScepHyBpox1, still remain unknown at the protein level.

### 3.4. Peculiar Conserved-Sequence Motifs of Hybrid B Heme Peroxidases

**Figure 4:**
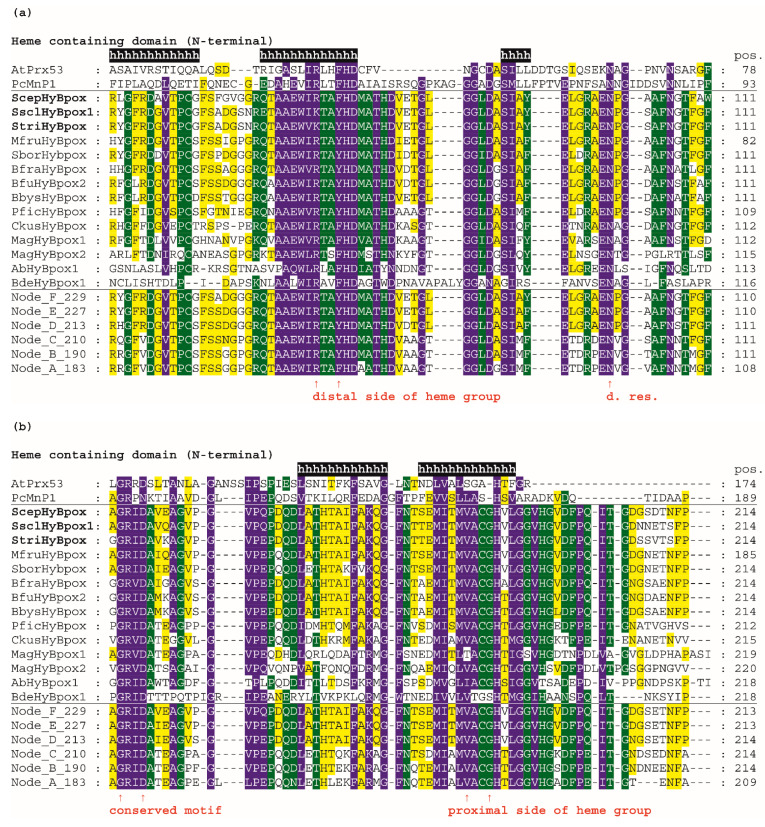

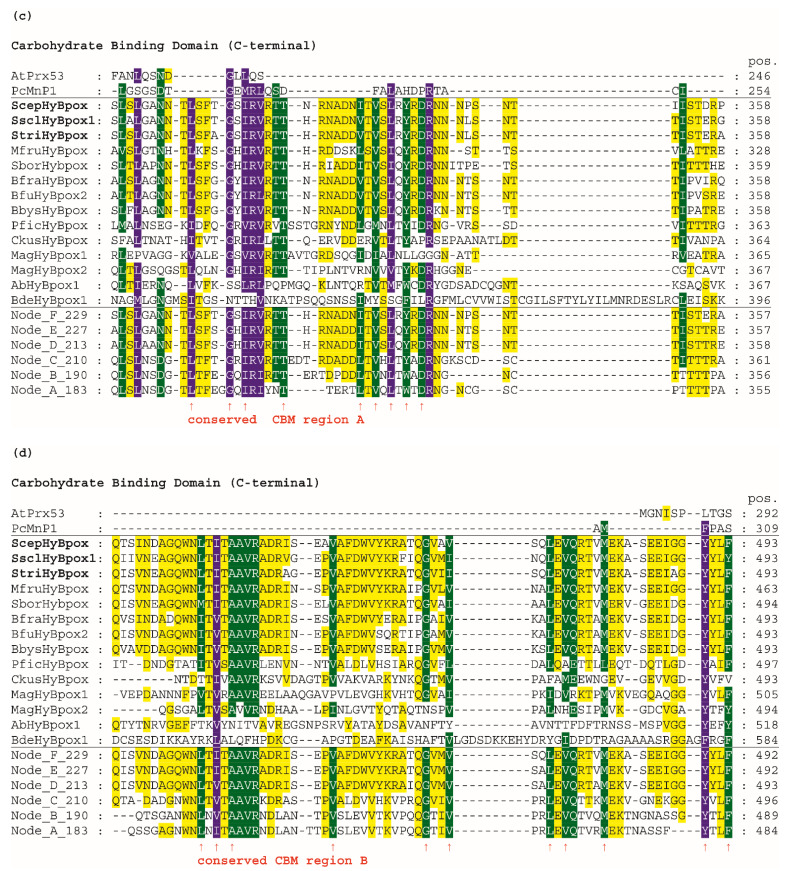
Multiple sequence alignment of 14 selected Hybrid B heme peroxidases with one typical plant-secretory and one typical fungal manganese peroxidase. Reconstructed protein sequences in 6 ancestral HyBpox nodes (labelled as in Figure 3) are given below. Only highly conserved regions are shown here. Complete sequence alignment is displayed in Figure S3. Conserved regions shown: (**a**) Distal side of prosthetic heme group; (**b**) Proximal side of prosthetic heme group; (**c**) Region A in carbohydrate-binding domain; (**d**) Region B in carbohydrate-binding domain. Secondary structure elements are from the 3D structure of PcMnp1. Abbreviations of sequence names are explained in Table S2, with corresponding accession numbers. Colour scheme: blue >90%, green >75%, and yellow >50% conservation.

Next, we analysed the conserved and unique features of the newly discovered subfamily named Hybrid B heme peroxidases and compared them with already well-known sequence features in corresponding amino-acid positions within the peroxidase–catalase superfamily. The complete multiple sequence alignments of selected Hybrid B heme peroxidases is presented in Figure S3, and the most important aspects are shown in in Figure 4.

For an easy overview, only 14 representative protein sequences from all resolved HyBpox subfamily clades of the evolutionary tree shown in Figure 3, including the newly discovered ScepHyBpox1, are shown. Additionally, sequences of six reconstructed ancestors in the labelled nodes A–F were also chosen for this alignment figure. For a general comparison, sequences from typical representatives of Families II and III (with already solved 3D structures) are given above the aligned Hybrid B peroxidases and their ancestors. Secondary-structure elements are derived from known 3D structures of fungal manganese peroxidase.

In the first two panels, the N-terminal heme peroxidase domain is presented, whereas in the last two panels, the C-terminal sugar-binding domain (CBM) that is typical among all known heme peroxidases solely for HyBpox proteins is shown. The highly conserved motifs on the distal (Figure 4a) and proximal (Figure 4b) sides of the prosthetic heme group can be observed here in detail and compared with the calculated sequences of particular ancestors. On the distal heme side, (a) we can mainly observe the presence of the essential catalytic triad involved in the heterolytic cleavage of the peroxide bond. These amino-acid residues are located in a long α-helix and defined previously as a typical **R-X-X-F-H-D** consensus sequence for all Family II and Family III heme peroxidases [36] that share a common ancestor with Hybrid B peroxidases (Figure 3). However, in Figure 4a we see remarkable diversity among Hybrid B peroxidases, which has deep evolutionary roots and is also seen in the sequences of corresponding ancestors in all the nodes presented (A–F). The most interesting variant is present in sequences of *S. sclerotiorum* and *S. trifoliorum* HyBpox as **K-T-A-Y-H-D.** The catalytic histidine responsible for the correct orientation and heterolytic cleavage of incoming peroxide is strictly conserved on the distal side. Additionally, the importance of the preceding arginine for the heterolytic cleavage of hydrogen peroxide was verified experimentally in distantly related CcP [37]. Although corresponding ariginine is present in almost all HyBpox sequences (Figure S3), the rare substitution to lysine is apparently only a recent evolutionary event in a few phytopathogenic fungi, which may yet have unknown impact on peroxidase reactivity. The last amino acid (aspartate) of this distal motif is supposed to be responsible for calcium-ion binding analogous with previously investigated single-domain heme peroxidases from Families II and III [38] and appears to be highly conserved among all sequenced Hybrid B peroxidases.

On the proximal side of heme, there is also a highly conserved motif that can be defined as **L/M-V-A-C-G-H-T/V** for all Hybrid B peroxidases and is also located in the α-helix. However, there are some differences also, including a deletion in Family-II and Family-III representatives (Figure 4b). This proximal motif appears to be more strictly conserved if compared with the distal side region of HyBpox and this is also the case for all other analysed members of the whole subfamily (not shown). Such a short motif, again including an essential histidine, can be considered as being essential for the fixed orientation of the prosthetic heme with iron in the catalytic centre of the peroxidases. Interestingly, however, the amino acid responsible for another calcium-ion binding in the proximal region [38] is not fully conserved and at least two different variants occur in the ancestors shown. This can have an impact on the capacity of calcium ions binding in the active centre of those Hybrid B peroxidases that have a non-polar amino-acid substitution in the corresponding position (Figure 4b and Figure S3). Most Family II and III single-domain peroxidases possess a rather conserved polar amino acid there. The stabilising effect of calcium ions was already verified experimentally in a HyBpox variant from *Magnaporthe oryzae* [2]. The obtained output can be compared with previous attempts for ancestral sequence reconstruction within the peroxidase–catalase superfamily, mainly focused on manganese and lignin peroxidases. In LiP of *P. Chryosporium,* a triple ancestral mutant on the proximal heme site led to slightly increased thermostability [39]. However, in Hybrid B peroxidases the corresponding region appears different and is highly conserved among all following descendants. A more recent reconstruction pointed out the occurrence of an exposed tryptophan, specifically in the lineage of LiP, that allows oxidation of nonphenolic lignin [40]. It can be deduced that this single substitution appeared long after segregation of HyBpox ancestors from common MnP, VP, and LiP ancestors, and it is therefore not present in any HyBpox ancestors (as clearly shown in Figure 4b).

In contrast to all manganese peroxidases, versatile, and lignin peroxidases, both known Hybrid B peroxidases, possess a unique CBM-containing domain. Details can be found in the panel of Figure 4c—namely, a highly conserved region in this C-terminal domain of HyBpox that is generally less conserved in comparison with the above described essential peroxidase-domain motifs. However, the unique sequence stretch **L/I/V-S-F-X-G-X-I/V-R-V/I-R-T-T** occurs solely in HyBpox sequences and is not present among any Family II or III peroxidases (also including generic peroxidases). To verify the relevance of this motif, we applied PSI-Blast (position-specific iterated) search in a non-redundant protein-sequence database and found that it is frequently present in numerous WSC-containing proteins (water soluble carbohydrate-binding domain) [41]. Some of the protein hits found in this way, mainly those from phytopathogenic fungi, probably also belong to the HyBpox subfamily, but there were also numerous protein sequences with such WSC domains that do not contain the preceding peroxidase domain. In the last panel presented in Figure 4d, there is a second conserved region of the CBM domain. We can observe here another unique motif **L/M/V-T-I/V-T/S-A-A-V-R-A-D-R-I** that has large differences only in the sequence of basal chytridiomycetous HyBpox and is completely absent in the sequences of Family II and III heme peroxidases. We again used PSI-Blast to reveal the relationships. The output again demonstrated its presence among numerous WSC-containing proteins and many of them without a preceding domain.

### 3.5. Typical Structural Folds in Hybrid B Heme Peroxidases

**Figure 5:**
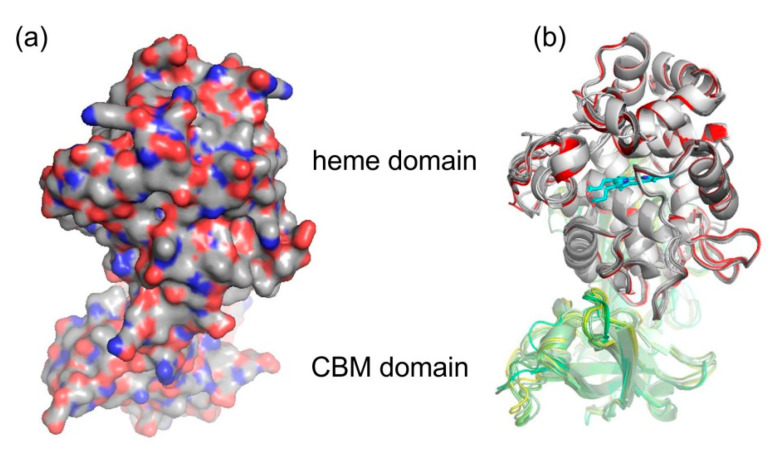
Structural presentation of a 3D model of *S. cepivorum* Hybrid B heme peroxidase in overlay with its direct ancestor (nodes A–F shown in Figure 3) reconstructed with AlphaFold 2 [25]. (**a**) surface presentation of the whole protein with usual colour scheme for amino acids; (**b**) domain organisation, colour scheme: red—peroxidase domain of ScepHyBpox1, grey—peroxidase domains of ancestors A-F, yellow—CBM domain of ScepHyBpox1, green—CBM domains of six ancestors, cyan—prosthetic heme group.

The spatial structure of yet completely unknown Hybrid B heme peroxidase from the phytopathogen *S. cepivorum* was modelled together with structures of its selected evolutionary ancestors in order to determine whether the typical heme-containing fold allowing peroxidase activity was preserved during the evolution of this subfamily, and how the second, non-heme domain is spatially related with the larger peroxidase domain. For this purpose we used the methodology of AlphaFold 2 [25] because no experimental 3D structure for any HyBpox representative is known yet, and AlphaFold is recommended particularly for those cases where the heme group is apparently essential for correct folding of the whole protein [25]. Although for Hybrid A heme peroxidase counterparts there are already available structures in the PDB database, e.g., 3RIV, [42] and they are phylogenetically very distantly related and are just single domain proteins. The result of neural-network modelling for ScepHyBpox1 as a compact two domain protein and for its direct ancestors is presented in Figure 5.

**Figure 6:**
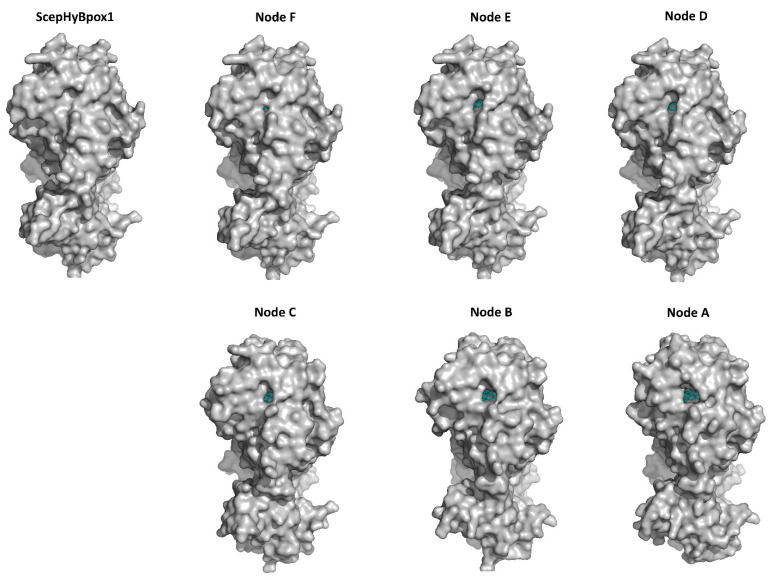
Presentation of 3D models for *S. cepivorum* Hybrid B heme peroxidase and its six ancestors (nodes A–F, shown in Figure 3) reconstructed with AlphaFold 2 [25]. The tunnel is seen most clearly in Node A and in all other structures it is narrowing. The prosthetic heme group at the end of the tunnel is coloured cyan.

The larger peroxidase domain that is mainly α-helical, as in the majority of other heme peroxidases from the whole peroxidase−catalase superfamily, e.g., Refs. [43,44,45] can be easily identified in the upper part of Figure 5. Although the overall sequence identity between HyBpox members and classical Family II and III peroxidases is in the rather low interval of 24–27%, the architecture of the heme domain is well preserved. The prosthetic heme group of ScepHyBpox1 is buried in the centre of this domain and accessible through tunnels from the protein surface. One such tunnel leading from the surface of the protein to the heme cavity on the distal side was modelled with CAVER [27], and the result is presented in Figure 6. This figure is supported by the obtained quantitative values for tunnel opening, length, bottleneck radius, curvature, and throughput, shown in Table 1. It can be followed that this tunnel was significantly changed during evolution. The curvature did not change significanlty and the length changed only slightly for all analysed structures. However, in ancestor A, the tunnel opening was rather broad and it was narrowed stepwise during evolution. Finally, in node E as well as in extant ScepHyBpox1, it was reduced down to 44% of the original ancestral size and is hardly discernible in Figure 6.

The smaller CBM domain occurs in contrast with a much lower portion of α-helical and more β-strand structural content that can be attributed mostly as β-sandwich. This typical double-domain structure of Hybrid B heme peroxidases represents a strictly conserved fold, observed also for the six reconstructed ancestral sequences in important evolutionary nodes (cf. Figure 3), which are presented in an overlay in Figure 5b and are separately displayed in Figure S4. Namely, this newly discovered fold was seen as remarkably conserved with the CBM domain always positioned closer to the proximal heme side of the peroxidase domain. This is true not only for recent ancestors that all belong to the phytopathogenic class of Leotiomycetes in nodes D (537AA long), E (536AA), and F (536AA) but also in deeper nodes. Reconstructed ancestral 3D structures in node C (539AA) represent sequences for common predecessors of Leotiomycetes and Dothideomycetes, and furthermore, node B (525AA) already includes a common ancestor shared with Basidiomycota class Agaricomycetes, where the same highly conserved double-connected domain architecture can still be observed. Finally, also node A (525AA)—as the last common ancestor of both main dikaryotic phyla, Ascomycota and Basidiomycota Hybrid B heme peroxidases were also reconstructed in the typical two domain composition with the same spatial orientation. Such detailed output of structural reconstruction in an evolutionary tree was impossible with classical homology modelling approaches because no suitable templates were available for the CBM domain (details not shown).

**Figure 7:**
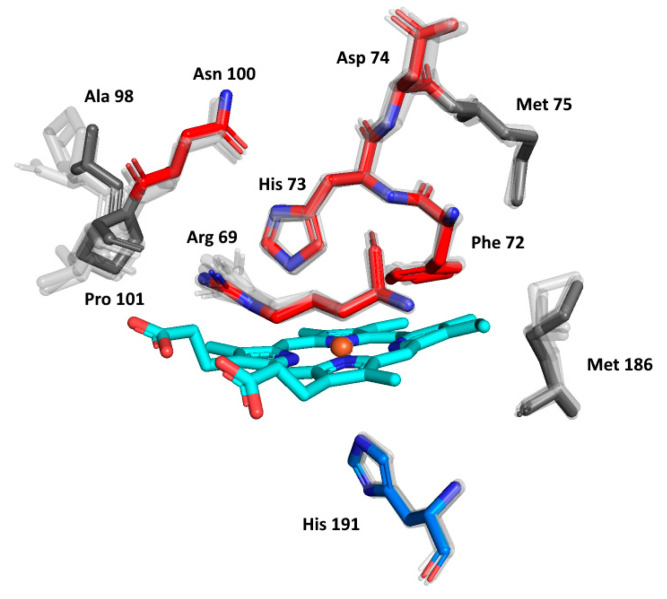
Structural presentation of a 3D model of the active centre in ScepHyBpox1 reconstructed with AlphaFold 2 [25]. Models of active centres for six ancestors from nodes A–F (cf. Figure 3) are also superimposed. Colour scheme: cyan—prosthetic heme group, red—residues involved in the catalytic machinery on the distal side, blue—proximal histidine.

The details of the reconstructed active centres of all the AlphaFold models were inspected in the presented phytopathogenic peroxidase and in its six ancestors in order to follow the fine details of the specific spatial orientation of the conserved amino acids allowing peroxidase reactivity. The output, again presented in the structural overlay, is shown in Figure 7. On the distal side of heme, the essential Arg69 and His73 that are responsible for the binding and conversion of the peroxidatic substrates can be seen. They are strictly conserved in their spatial orientation. There is some variability in the neighbouring position, represented by Phe72 (as seen in Figure 4a), but the spatial orientation of this amino acid can also be expected to be conserved. Moreover, Asn100, which builds important H-bridges in the active centre of, e.g., manganese peroxidases, is also perfectly conserved, but the neighbouring amino acids reveal some variability in Hybrid B heme peroxidases. Asp74, allowing the binding of calcium ions [38] near the heme group, is also fully conserved. The proximal side of the prosthetic heme group is not directly involved in catalysis in, e.g., Hybrid A heme peroxidases, but its conserved architecture is responsible for the correct orientation of the whole active centre for efficient catalytic turnover [36]. As can be observed in the lower part of Figure 7, essential His 191, coordinating the position of the heme iron atom, is also strictly conserved in all functional heme peroxidases, although it exhibits some variations (such as the position of Met 186). The situation here is different from the exceptional rearrangement of the proximal side observed in lignin peroxidases and their ancestors [46], and we can expect that Hybrid B peroxidases are more similar to, e.g., horseradish peroxidase on the proximal heme side that represents a different evolutionary line to LiP.

In principle, all above presented structural figures clearly show that the N-terminal heme-binding peroxidase domain was highly conserved during its entire evolutionary history, thus allowing essential and efficient peroxidase activity. On the other hand, there is additional variability, which is not involved in peroxidation cycles, in the additional CBM-binding domain on the C-terminus of Hybrid B peroxidases.

**Figure 8:**
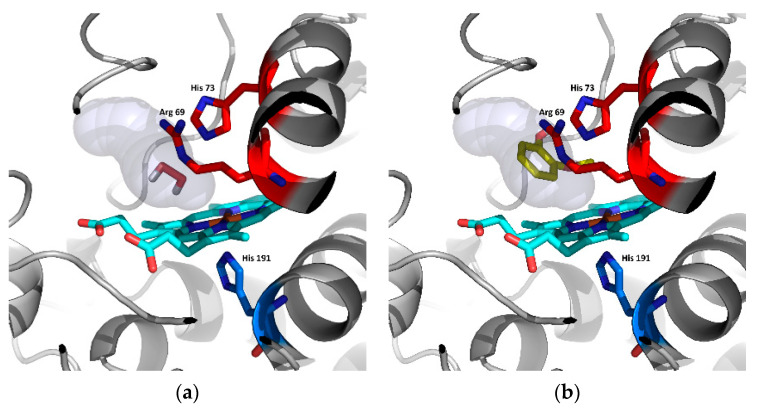
Analysis of potential substrates binding in the active centre of Hybrid B heme peroxidase—the ancestor from node A using AutoDock Vina [28]. (**a**) Docking of hydrogen peroxide and (**b**) docking of guaiacol, cyan—prosthetic heme group.

Finally, two potential substrates were docked in the active centres of all of the models presented here. Hydrogen peroxide is the first and most common substrate reduced in the active centres of all peroxidases. The second potential substrate—guaiacol—is used by a large group of evolutionary-related peroxidases as an electron acceptor for reactive enzyme intermediates. Results are presented in Table 2 and Figure 8.

Table 2 shows that the binding affinity of hydrogen peroxide is almost the same for ScepHyBpox1 as for all the ancestors analysed herein. This means that hydrogen peroxide can surely diffuse through the suggested tunnel from Figure 6 to the distal heme cavity and bind there for its subsequent reduction. This is the basic assumption for an enzyme functioning as a peroxidase. On the other hand, there is a significantly worse binding affinity for guaiacol in ScepHyBpox1 in comparison with all ancestral peroxidase models. The best binding affinity reveals the ancestor from Node A and this corresponds well with the rather large tunnel opening and the very good tunnel throughput presented in Table 1 and seen in Figure 6.

In Figure 8, the spatial relationships are depicted for the docking of the two described substrates in ancestral node A. It is likely that hydrogen peroxide is easily cleaved in this active centre, and guaiacol can also be oxidised by ancestor A, but this oxidation is very unlikely due to the narrow tunnel opening and low throughput for extant ScepHyBpox1, which may have evolved another mechanism for the oxidation of phenolic compounds.

## 4. Discussion

We demonstrated that in a typical secreted heme-peroxidase representative from a phytopathogenic fungus, namely ScepHyBpox1 and its ancestors, there exists an important novel and still rather undiscovered heme peroxidase subfamily that may have an important function for pathogenic fungal cells in their defence against oxidative bursts caused by the host plant. Corresponding mRNA is produced rather constitutively and is translated into a unique two-domain protein representing the sole secreted peroxidase of this filamentous fungus. This fact underlines its importance for the survival of the described phytopathogen and probably also for related pathogenic fungi from the whole class of Leotiomycetes during infection cycles and when attacking plant tissues. Numerous Hybrid B heme peroxidase sequences obtained from different fungal families of both Ascomycota and Basidiomycota were systematically collected for phylogenetic purposes. Even the presence of corresponding Hybrid B heme peroxidase sequences that are supposed to be an early diverging lineage of the whole fungal kingdom were detected among Chytridiomycota. All representatives of the newly discovered peroxidase subfamily presented here reveal surprisingly high overall sequence similarity which is mainly localised in the larger heme-peroxidase domain on the N-terminus of the protein, irrespective of their trophic levels in various ecosystems. The C-terminal domain that is directly fused with the larger α-helical peroxidase domain is quite unusual for all previously described heme peroxidases, but it is also present in all of the known deep ancestors of this subfamily in nearly the same orientation where the CBM-containing domain is located near the region of the proximal side of heme. The proposed “hybrid“ character of the proteins analysed herien is concealed in this unique double-domain architecture and apparently has deep evolutionary origins among early-diverging aquatic fungal lineages. This observation is in noticeable in constrast to, e.g., lignin peroxidases among early-diverging fungi that have not yet been described. In contrast, the presence of these flexible enzymes among Chytridiomycota before their presence among Dikarya indicates that, in addition to their basic protective function against reactive oxygen species, they may have been involved in the ancient extraction of useful nutrients from early land-colonising plants and their algal relatives [32]. In this way, Hybrid B heme peroxidases represent a unique fusion product that posed certain evolutionary advantages and a kind of positive selection for the mostly pathogenic fungi involved in long-term interactions with land plants. These unique, hybrid fusion proteins may eventually become a promising target for future biotechnological design focused on compact, bifunctional proteins within a rather variable but tight fusion, and which may serve as a target for rational protein engineering to yield new functionalities.

## 5. Conclusions

In this study, a new type of Hybrid B heme peroxidase was detected and investigated on the proteomic level as the sole peroxidase in the secretome of an important phytopathogenic fungus. The reconstruction of its broad phylogenetic relationships in corresponding peroxidase (sub)families reveals its importance as a model protein representing numerous, related heme peroxidases. Its unique two-domain architecture was modelled, and a typical tunnel leading to the distal heme cavity was resolved by the advanced method of structural modelling methodology represented by AlphaFold and CAVER. The performed docking of two potential substrates must be verified in a highly purified sample of this or a related Hybrid B heme peroxidase. The observed Hybrid B heme and CBM-fusion proteins are now attractive targets for future functional and engineering studies.

## Figures and Tables

**Table 1:** Comparison of the tunnels with the heme cavity in analysed peroxidases calculated by Caver software [27].

Protein	Throughput	Tunnel Opening	Bottleneck Radius	Length	Curvature
ScepHyBpox1	0.58	1.4	1.2	12.6	1.2
Node F	0.56	1.5	1.1	13.7	1.2
Node E	0.62	1.9	1.2	13.0	1.2
Node D	0.76	1.8	1.5	9.2	1.1
Node C	0.60	1.9	1.0	11.6	1.1
Node B	0.64	2.2	1.1	11.3	1.1
Node A	0.68	2.5	1.1	11.7	1.2

**Table 2:** Potential substrate binding in active centres of all modelled heme peroxidases, calculated using Autodock Vina software [28].

Protein	Binding Affinity (kcal/mol)	
	Hydrogen Peroxide	Guaiacol
ScepHyBpox1	−2.8	0.8
Node F	−2.4	−2.2
Node E	−2.4	−3.8
Node D	−2.3	−5.0
Node C	−2.6	−1.6
Node B	−2.9	−3.5
Node A	−2.2	−5.1

## Data Availability

Data are contained within the article and Supplementary Materials.

## Supplementary Materials

The following are available online at https://www.mdpi.com/article/10.3390/biology11030459/s1, Figure S1: DNA electrophoresis showing comparison between genomic DNA fragments and cDNA fragments of ScepHyBpox1 (2 samples: induced with H_2_O_2_ and non-induced); Figure S2: Annotated fragmentation spectra belonging to peptides from ScepHyBpox1; Figure S3: Multiple sequence alignment of selected Hybrid B heme peroxidases (complete in fasta format); Figure S4: Presentation of separated 3D models of ScepHyBpox1 and its six ancestors as obtained from AlphaFold; Table S1: DNA primers used for the amplification and sequencing of ScepHyBpox1 cDNA; Table S2: Overview of abbreviations used and accession numbers for aligned peroxidase sequences; Table S3: All detected extracellular proteins in the secretome of *S. cepivorum*; Table S4: Identification details, quantification, and statistical analysis of ScepHyBpox1 in obtained secreted and cellular fractions.
