# Supplementary material for: Deep Insights into the Specific Evolution of Fungal Hybrid B Heme Peroxidases
Source: Biology (Basel). 2022 Mar 17;11(3):459. doi: 10.3390/biology11030459 (PMC8945051; doi:10.3390/biology11030459)
Supplement: Supplementary file 1 [file biology-11-00459-s001.zip › Table S1.pdf]

Tab.S1 DNAPrimers used in this study

Primer abbreviation	Sequence (5’ → 3’)	Description
ScepHyBpoxRT-F1	ATGAAGGCGCTATCTTTGATCAGT	*ScepHyBpox1* specific gene primers designed in this work
ScepHyBpoxRT-R1	AAGTCCGAGGAGATGTTGTGTTCT	
ScepHyBpoxRT-R2	GTCCTCCAAGACCAGTCTCTACAT	
