# Supplementary figures and images for: Deep Insights into the Specific Evolution of Fungal Hybrid B Heme Peroxidases
Source: Biology (Basel). 2022 Mar 17;11(3):459. doi: 10.3390/biology11030459 (PMC8945051; doi:10.3390/biology11030459)

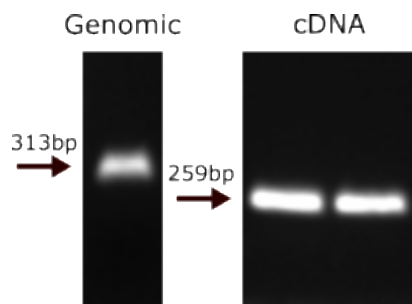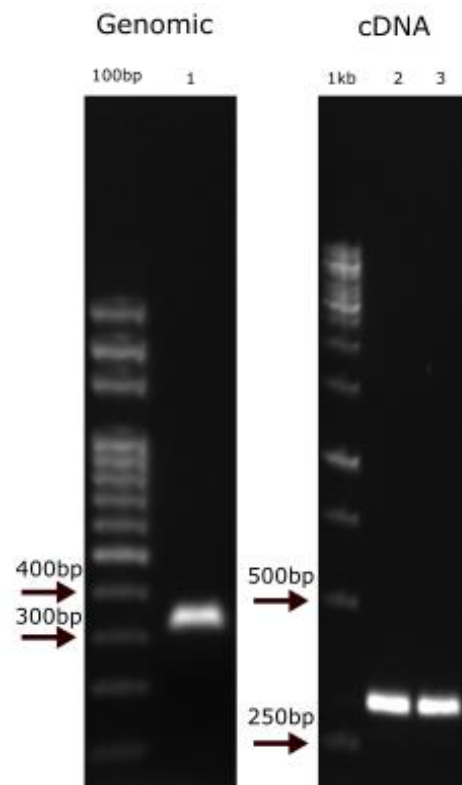

Supplement: Supplementary file 1 [file biology-11-00459-s001.zip › Figure S1 two.pdf]

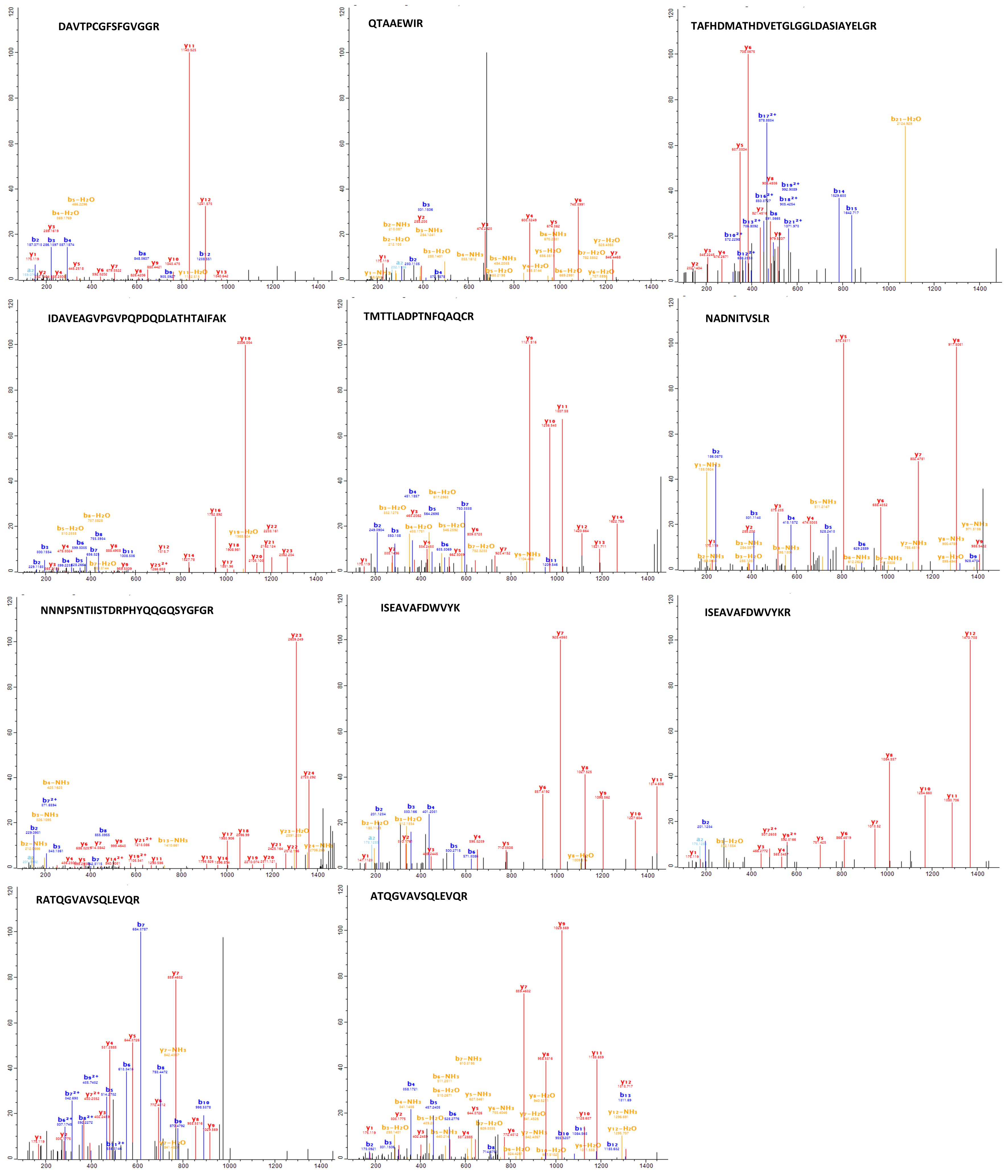

Supplement: Supplementary file 1 [file biology-11-00459-s001.zip › Figure S2 MS fragmentation spectra ScepHyBpox1.tif]

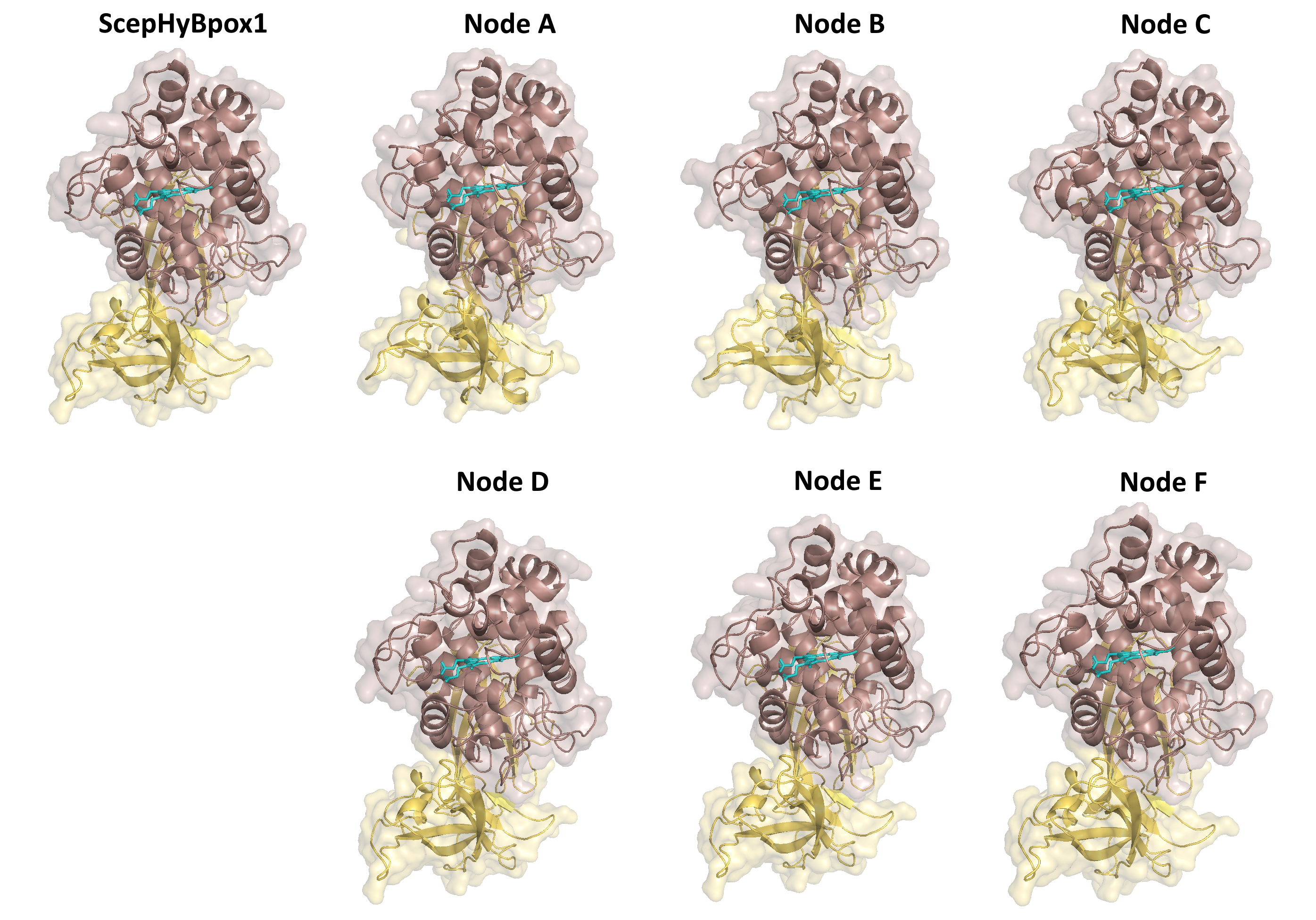

Supplement: Supplementary file 1 [file biology-11-00459-s001.zip › Figure_S4_structures AlphaFold.png]
